# Advancing hyperspectral imaging techniques for root systems: a new pipeline for macro- and microscale image acquisition and classification

**DOI:** 10.1186/s13007-024-01297-x

**Published:** 2024-11-11

**Authors:** Corine Faehn, Grzegorz Konert, Markku Keinänen, Katja Karppinen, Kirsten Krause

**Affiliations:** 1 Department of Arctic and Marine Biology, The Arctic University of Norway, 9037 Tromsø, Norway; 2 Department of Life Technologies, University of Turku, 20014 Turku, Finland; 3 Department of Environmental and Biological Sciences, University of Eastern Finland, 80130 Joensuu, Finland; 4 Center for Photonics Sciences, University of Eastern Finland, 80110 Joensuu, Finland; 5 Arctic Centre for Sustainable Energy, The Arctic University of Norway, 9037 Tromsø, Norway

**Keywords:** Graminoid, Root phenotyping, VNIR SNAPSCAN, Image analysis, Biomass estimation, Root-soil interface

## Abstract

**Background:**

Understanding the environmental impacts on root growth and root health is essential for effective agricultural and environmental management. Hyperspectral imaging (HSI) technology provides a non-destructive method for detailed analysis and monitoring of plant tissues and organ development, but unfortunately examples for its application to root systems and the root-soil interface are very scarce. There is also a notable lack of standardized guidelines for image acquisition and data analysis pipelines.

**Methods:**

This study investigated HSI techniques for analyzing rhizobox-grown root systems across various imaging configurations, from the macro- to micro-scale, using the imec VNIR SNAPSCAN camera. Focusing on three graminoid species with different root architectures allowed us to evaluate the influence of key image acquisition parameters and data processing techniques on the differentiation of root, soil, and root-soil interface/rhizosheath spectral signatures. We compared two image classification methods, Spectral Angle Mapper (SAM) and K-Means clustering, and two machine learning approaches, Random Forest (RF) and Support Vector Machine (SVM), to assess their efficiency in automating root system image classification.

**Results:**

Our study demonstrated that training a RF model using SAM classifications, coupled with wavelength reduction using the second derivative spectra with Savitzky-Golay (SG) smoothing, provided reliable classification between root, soil, and the root-soil interface, achieving 88–91% accuracy across all configurations and scales. Although the root-soil interface was not clearly resolved, it helped to improve the distinction between root and soil classes. This approach effectively highlighted spectral differences resulting from the different configurations, image acquisition settings, and among the three species. Utilizing this classification method can facilitate the monitoring of root biomass and future work investigating root adaptations to harsh environmental conditions.

**Conclusions:**

Our study addressed the key challenges in HSI acquisition and data processing for root system analysis and lays the groundwork for further exploration of VNIR HSI application across various scales of root system studies. This work provides a full data analysis pipeline that can be utilized as an online Python-based tool for the semi-automated analysis of root-soil HSI data.

**Supplementary Information:**

The online version contains supplementary material available at 10.1186/s13007-024-01297-x.

## Introduction

Root-soil interactions in the rhizosphere are vital for plant and ecosystem health. The rhizosheath, where root exudates cause soil particles to adhere, plays a key role in these interactions by enhancing root water retention and plant resilience against environmental stresses [1]. Robust root systems, together with their rhizosheath, improve water and nutrient uptake, stabilize soil structures, and prevent soil erosion [2, 3], while also protecting plants against soil-borne diseases [4]. Despite their importance, studying these interactions poses significant challenges due to the hidden and complex nature of root systems below the soil surface [5].

The use of rhizoboxes or rhizotrons—thin soil-filled chambers with transparent observation windows—has enabled non-destructive surveillance and imaging of root development, providing valuable insight into key root responses to different soil and environmental conditions over time [6–8]. Innovative methods have been developed to integrate these set-ups with luminescence-based reporters allowing for the examination of root architecture and gene expression in soil-grown roots [9]. Additionally, the use of transparent tubes equipped with cameras can be inserted into the soil, enabling the capture of 360° images for in-situ monitoring of root development in the field [10, 11]. Hyperspectral imaging (HSI) offers new possibilities for studying these complex interactions in greater detail. HSI captures a broad spectrum of electromagnetic radiation beyond the visible range, providing unique spectral signatures for each pixel. This technology not only extends our visual perception but can also provide qualitative and quantitative information on the physiological state and chemical composition of roots and the surrounding soil without extensive chemical analyses [12].

The use of HSI for root phenotyping of soil-grown plants is a relatively new approach. Traditionally, many HSI approaches have focused on aboveground data to indirectly assess root health status [13, 14], but recent studies have expanded its direct application to root imaging. Bodner et al. [12] demonstrated HSI’s capability to detect the radial composition and decomposition dynamics of root axes using spectral signatures in the 1000–1700 nm range, which can be combined with RGB imaging to determine root structural traits [5]. Additionally, VNIR HSI has been utilized to predict lead stress levels in oilseed rape leaves and roots [15], classify growth years of Kudzu roots [16], and distinguish between leaf mold and soil in the rhizosphere [17]. VNIR HSI has also recently been used to monitor the roots of peanut and sweet corn under varying drought conditions, with this data available in a publicly accessible HyperPRI dataset [18]. This dataset was useful to develop models that predict root and soil water potentials, enhancing our understanding of drought tolerance and recovery in crops [18, 19]. The availability of such data, along with detailed acquisition methodologies and spectral signatures, is crucial for advancing research on rhizosphere processes. When integrated with other analytical techniques, such as physiological phenotyping and functional genomics, HSI can be a powerful tool to complement the genotype-to-phenotype gap as part of a comprehensive research approach [20, 21].

While traditional HSI systems, such as linescan or pushbroom, rely on mechanical scanning, requiring linear movement of either the object or the camera to capture the complete and spectral range, snapshot cameras capture the entire field of view without the need for spatial scanning. The SNAPSCAN camera (imec, Leuven, Belgium) merges linescan and snapshot imaging principles using on-chip filter technology, which simplifies the system assembly and enhances its usability for root phenotyping. This camera, adaptable for use with front optics or microscope integration, has shown promise in various agricultural contexts, including plant species classification [22], estimating fruit maturity [23], and outdoor weed detection [24]. Its application in microscopy has primarily been in biomedical contexts [25–27], but to our knowledge, it has not yet been used in root phenotyping. Despite these valuable advances, numerous challenges must be addressed before the utilization of HSI to analyze root systems will be comparable to its application in other areas of plant research.

Although the aforementioned articles have demonstrated the SNAPSCAN camera’s versatility, the quality of the HSI data depends on user-selected configurations and acquisition settings, a critical aspect that has received limited attention in the existing literature. Manually adjusted settings, including the distance between the sample and lens, lens aperture, and critical software parameters such as time delay integration (TDI) pixel step, pixel binning, and integration time, affect the duration of image acquisition, spatial resolution, and the signal-to-noise ratio. These factors are essential because they directly influence the quality of the acquired image data. Various settings can be adjusted to balance between acquiring high-quality images and faster image capture, however, the subsequent image processing steps are also fundamental for refining the data. These steps typically involve eliminating dead pixels, selecting specific regions of interest (ROI), enhancing spectral features through pre-processing, and compressing the image to retain only pertinent information [28]. While the SNAPSCAN software automatically performs some pre-processing steps, enabling immediate exploration of the data, the choice of further data processing is objective dependent. Consequently, HSI data processing often necessitates tailored solutions adapted to the specific experimental settings.

To effectively utilize HSI data for investigating root systems and the biochemical composition in root-soil interactions, it is crucial to fully comprehend the capabilities and limitations of this technology. Therefore, this study aimed to investigate the techniques of image acquisition and data processing for evaluating plant root systems using VNIR SNAPSCAN technology across three distinct dimensional scales: from an overview scale that captures the entire rhizobox to a microscopic scale focusing on individual roots using a stereomicroscope. Since different plant root traits such as diameter, density, and rhizosheath composition may present unique challenges for HSI, we selected three graminoid species with distinct root system characteristics: *Deschampsia flexuosa, Eriophorum vaginatum,* and *Anthoxanthum odoratum*. All three species are well-adapted to survival in nutrient-poor, acidic soils, and the low temperatures of subarctic ecosystems, but have different root growth strategies [29–31]. Our analysis assesses whether the SNAPSCAN camera can distinguish root traits of different species across varied imaging scales to explore the applicability of HSI in studying root adaptations to harsh environmental conditions. We employed a methodical strategy that utilized a small set of samples to distinguish between roots, soil, and the root-soil interface by varying image acquisition settings and evaluating the data through image classification and processing techniques. Additionally, we discuss potential challenges associated with the use of the SNAPSCAN camera and provide recommendations regarding the technical framework for future experimental set-ups focused on analyzing root-soil interactions.

## Materials and methods

### Plant material and rhizobox cultivation

Whole, intact plants including the root systems of the grass *D. flexuosa* and the sedge* E. vaginatum* were collected from a natural peat bog at Håkøybotn, Tromsø, Norway (69° 63’N, 18° 78’E) in late summer of 2021. The grass *A. odoratum* was collected from a previously revegetated urban site at Holt, Tromsø, Norway (69° 65’N, 18° 91’E). *D. flexuosa* and *A. odoratum* are true grasses, which have fibrous and highly branched perennial root systems, while *E. vaginatum* has thick, unbranched annual root systems [30]. All plants were propagated vegetatively in a greenhouse (15 °C, 18 h light, and photosynthetic photon flux density (PPFD) of 200 μmol m^–2^ s^–1^) at the Climate Laboratory in Holt, Tromsø, Norway.

**Fig. 1:**
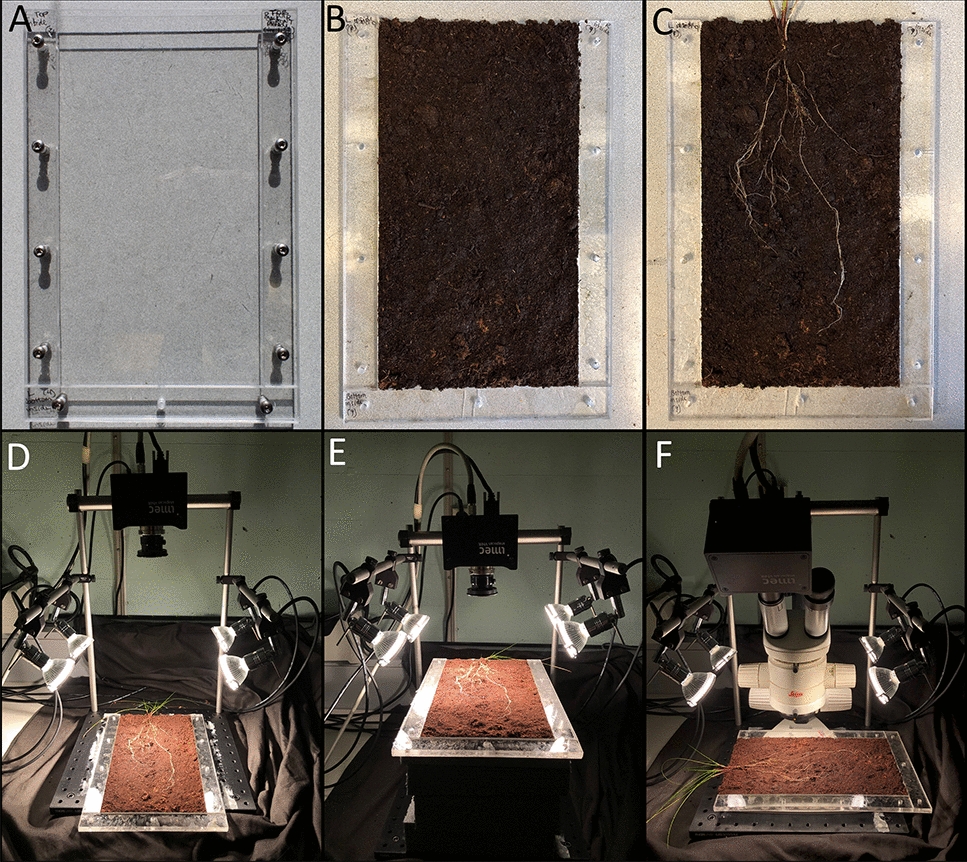
Experimental set-up for root growth and image acquisition configurations. **A** Empty rhizobox, **B** rhizobox pre-filled with peat soil and (**C**) opened rhizobox with root system on the soil surface. **D** Configuration 1 (CONF1) for SNAPSCAN VNIR imaging including the camera equipped with a Schneider Kreuznach Apo-Xenoplan lens mounted on a frame connected to the imaging stand with a 34.5 cm working distance (WD) between the camera lens and the sample surface. **E** CONF2 using the same set-up as CONF1 with a WD of 14 cm. **F** CONF3 used the camera with a 0.5x C-mount lens adapter mounted to a stereomicroscope. The WD between the stereomicroscope lens and sample surface varied between 10 and 14 cm according to the different magnifications

Individual rhizoboxes consisted of two clear plexiglass panels (20 cm × 30 cm × 0.15 cm), two plexiglass side frames (2.5 cm × 27.5 cm × 0.6 cm) and a bottom plexiglass frame (2.5 cm × 20 cm × 0.6 cm) in between giving a spatial volume of 247.5 cm^3^ (Fig. 1A). The back panel, two side frames, and bottom frame were glued together before the rhizoboxes were filled with pre-moistened peat soil (Fig. 1B). The roots of all graminoids were cut to approximately 4 cm in length and transplanted individually in rhizoboxes at a depth of 1 cm below the soil surface (Fig. 1C). Then the front panel was fastened with screws and hex nuts allowing easy removal of the front panel for later imaging. The rhizoboxes were placed in opaque plastic bags to block light entry and positioned at a 45-degree angle, with the front panel facing down to promote root growth along this imaging plane. Throughout the experiment, the rhizoboxes were kept in the same greenhouse conditions described above and watered frequently to maintain high moisture levels.

### HSI system set-up and acquisition parameters

Hyperspectral imaging (HSI) was performed using the VNIR SNAPSCAN camera (imec, Leuven, Belgium). The SNAPSCAN sensor has a spectral resolution of 150 bands in the 470–900 nm wavelength range, and a spatial resolution of up to 3650 × 2048 pixels (7 Mpixels RAW per band). The sensor frame rate has a maximum of 340 fps. Four halogen lamps (2000 K) equipped with diffusers and 11.83 V and 6.72 A of power were used for illumination. Lamps were connected to the imaging stand provided by imec, consisting of a viewing stage and frame to hold the camera and lamps. Hyperspectral images were taken at three different configurations (CONFs), where CONF1 (Fig. 1D) and CONF2 (Fig. 1E) utilized the Schneider Kreuznach Apo-Xenoplan lens (f2.0, C-mount, focal length 24 mm, provided by imec) with different distances between the sample and lens, and CONF3 (Fig. 1F) utilized a 0.5x C-mount lens adapter in the ocular of a Leica MS5 stereomicroscope (Leica Microsystems, Wetzlar, Germany), used with 0.63x, 1.6x, and 4x magnification. The front panel of the rhizobox was removed for imaging in every configuration to avoid transmittance effects of the plexiglass panel. The acquisition parameter used for each configuration are listed in Table 1.

**Table 1:** Acquisition parameters tested for each of the three configurations for the VNIR SNAPSCAN camera

Settings										# images used for pre-classification		# images used to train machine learning model	Total # images for classification
CONF	Lens	WD	CS	CD	Bin	TDI	MPB	Lens aperture / magnification	IT	SAM	K-Means		
**1**	Schneider Kreuznach Apo-Xenoplan lens	34.5 cm	764 × 1024	115 × 155 mm	2 × 2	3	2.5	f/2.8	4–7 ms	–	–	–	18
								f/4.0	10–15 ms	–	–	–	18
								f/5.6	15–20 ms	12	12	6	30
**2**	Schneider Kreuznach Apo-Xenoplan lens	14 cm	1528 × 2048	49 × 66 mm	1 × 1	1	0	f/8.0	18 ms	6	6	–	8
								f/11.0	18 ms	9	9	9	16
							2.5	f/11.0	18 ms	3	3	–	3
						5	0	f/11.0	18 ms	3	3	–	3
**3**	0.5X lens + stereomicroscope	~ 14 cm	800 × 800	15 × 15 mm	1 × 1	1	0	0.63X	10 ms	9	9	9	9
		~ 11 cm		8 × 8 mm				1.6X	10 ms	9	–	–	9
		~ 10 cm		5 × 5 mm				4X	30 ms	9	–	–	9
								Total images:		60	42	24	123

*CONF* configuration, *WD* working distance, *CS* cube shape, *CD* cube dimension, *Bin*. binning, *TDI* time delay integration, *MPB* max. pixel blur, *IT* integration time, *SAM* spectral angle mapper

The hyperspectral images were collected using the HSI SNAPSCAN (V1.8.1.1) software. The white reference image was acquired by scanning the white reference target (provided by imec) at the settings used for sample acquisition with reflectance set to 95%. The dark reference image was acquired using the built-in mechanical shutter. Due to the time-intensive nature of HSI, capturing images of many biological replicates under different settings on the same day was not feasible. Thus, for CONF1, two biological replicates per species were imaged at five time points over the course of twenty days. In CONF2, two biological replicates per species were imaged at two timepoints over eight days. For CONF3, three technical replicates of a single biological replicate for each species were imaged on one day. The total number of images are listed in Table 1.

### Hyperspectral image pre-processing, classification, and data reduction

**Fig. 2:**
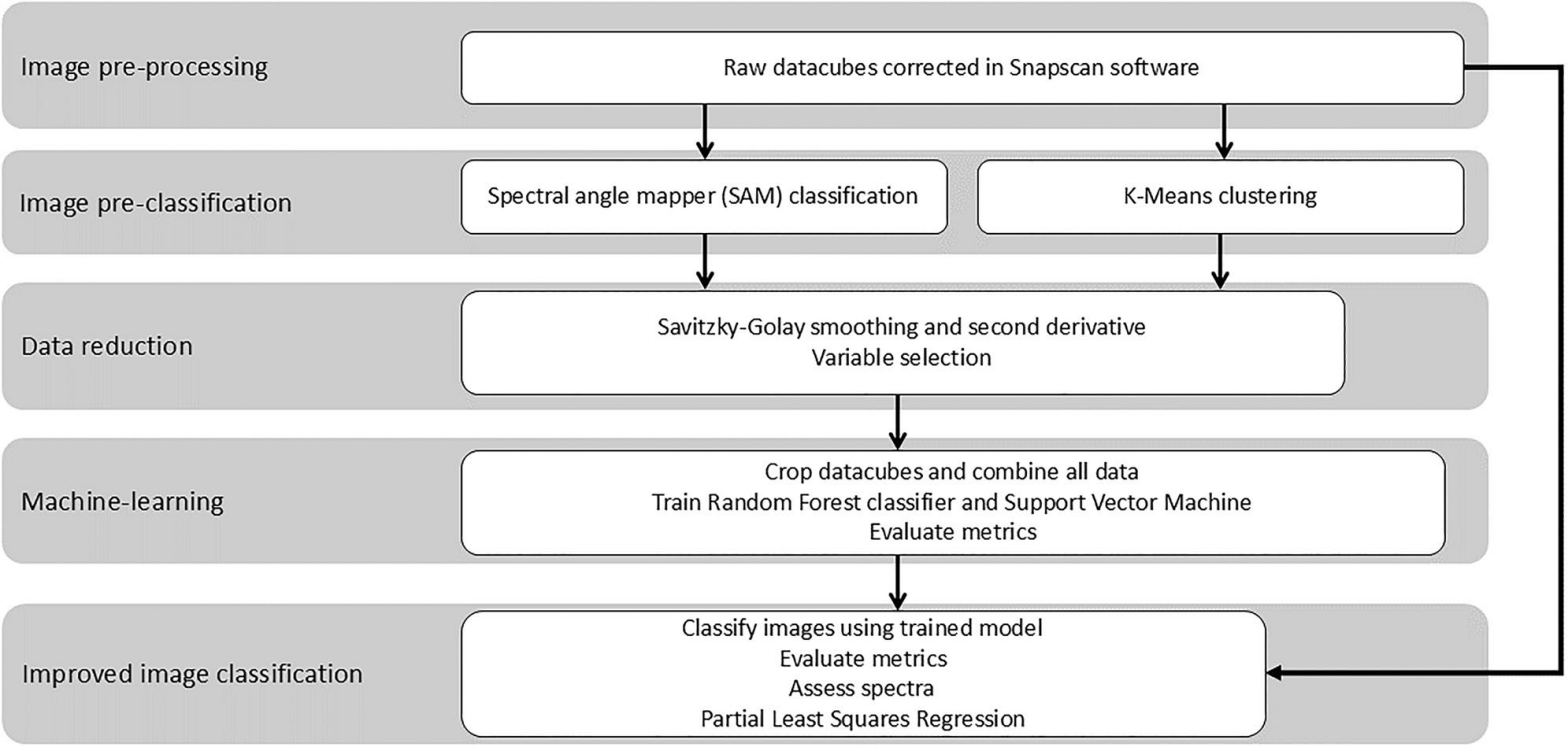
Data processing workflow for hyperspectral image analysis. Image pre-processing and SAM classifications were carried out in the HSI SNAPSCAN software. All other steps were carried out in Python

An overview of the data processing workflow is represented in Fig. 2. Reflectance corrections for the white and dark reference were carried out automatically in the HSI SNAPSCAN software. After this, each image was classified using a supervised and an unsupervised classification method. The supervised Spectral Angle Mapper (SAM) classifications were also carried out in the HSI SNAPSCAN software. Three different regions of interest (ROI) were manually selected for root, soil, and the root-soil interface, with a minimum of 100 pixels per ROI. These ROIs were used to run the SAM classification with a spectral angle of 10 degrees. All remaining data processing was carried out in Python v3.2. Unsupervised K-Means clustering was carried out using the *Spectral Python* (*SPy*) v0.23.1 module [32]. After the raw datacube import, pixels with a reflectance value above 1 (overexposed) were set to 0. K-Means clustering was run with three clusters and a maximum of 20 iterations. Each classification method gave a classified image with three classes and the corresponding average spectral reflectance values for each class.

The spectral data for each of the classification methods were imported using the *pandas* v1.4.2 module [33]. To reduce the large number of wavelengths to a smaller number of representative variables, Savitzky-Golay (SG) smoothing [34] using the second derivative was applied using the *SciPy* v1.7.3 module [35]. The second derivative emphasizes small spectral variations and removes some residual scattering effects, mainly additive effects and linear baseline shifts [36], thus facilitating the selection of the most informative bands from the spectrum. A comparison of window sizes was applied to the second derivative of the root spectra data to reduce the effect of noise but maintain the most important spectral information. Using the chosen window size, the average of the second derivative was taken across each root spectra data set to identify the most informative bands.

### Implementation of machine learning for image classification and biomass estimation

The acquisition setting that consistently produced the best classifications across all three species was chosen to construct a robust algorithmic model for each configuration. At least two images per species were used to train each model to ensure that each species was equally represented. Raw datacubes and their corresponding classified images were imported using the *SPy* and *Pillow* (*PIL*) v9.0.1 [37] modules. For each configuration, two models were generated: one trained on the SAM classifications and the other trained on the K-Means clusters. The datacubes and class images were cropped to a region of 300 × 300 pixels at the root-soil interface, encompassing all three classes. These datasets were reshaped into two-dimensional dataframes, where each pixel represented a row, and the selected bands served as columns. Each pixel was assigned to one of the three classes based on the respective classified image. All data for each model were consolidated into a single dataframe, and rows containing unlabeled pixels from the SAM classification were excluded.

*RandomForestClassifier* (RF) and *SVC* (Support Vector Machine Classification, SVM) from the *Scikit-learn* v1.0.2 module [38] were implemented for machine learning. The dataframes were randomly split into a training set (80%) and a testing set (20%) using a random state of 0 and either a fixed number of estimators (50) for the RF model, or a linear kernel for the SVM model. After being fitted using the assigned classes, the models were used to predict the testing set. Models were evaluated based on the *classification_report*, *confusion_matrix*, and *accuracy_score* metrics from the *Scikit-learn* module. The models developed were used to classify the root, soil, and interface regions across all rhizobox images in each respective configuration and evaluated based on the same metrics stated above. The spectral signatures of each class were used to assess the differences between the configurations, acquisition parameters, and species. A Partial Least Squares (PLS) regression [39] was used to evaluate the correlations between the predicted classes with each species and configuration.

For the model predicted images in CONF1, the number of pixels in each class were converted to percentages using the *PIL* and *webcolors* v1.13 modules. The dimensions of the images (115 × 155 mm) were then used to calculate the estimated biomass area for each class. The scripts for data analysis are available from GitHub (see Availability of Data and Materials).

## Results

### Classification challenges of the root, soil, and root-soil interface in different configurations

Three regions of interest (ROI), determined by grouping pixels with similar spectral features, were utilized by two classification methods, Spectral Angle Mapper (SAM) classifications and K-Means clustering, to distinguish root, soil, and root-soil interface regions in images of root systems grown in rhizoboxes. The interface class, designed to include inorganic soil particles and live root hairs within the organic mucilaginous matrix of the rhizosheath, was crucial to clearly differentiate between root and soil classes due to its heterogeneity and the spatial resolution limits of the camera.

**Fig. 3:**
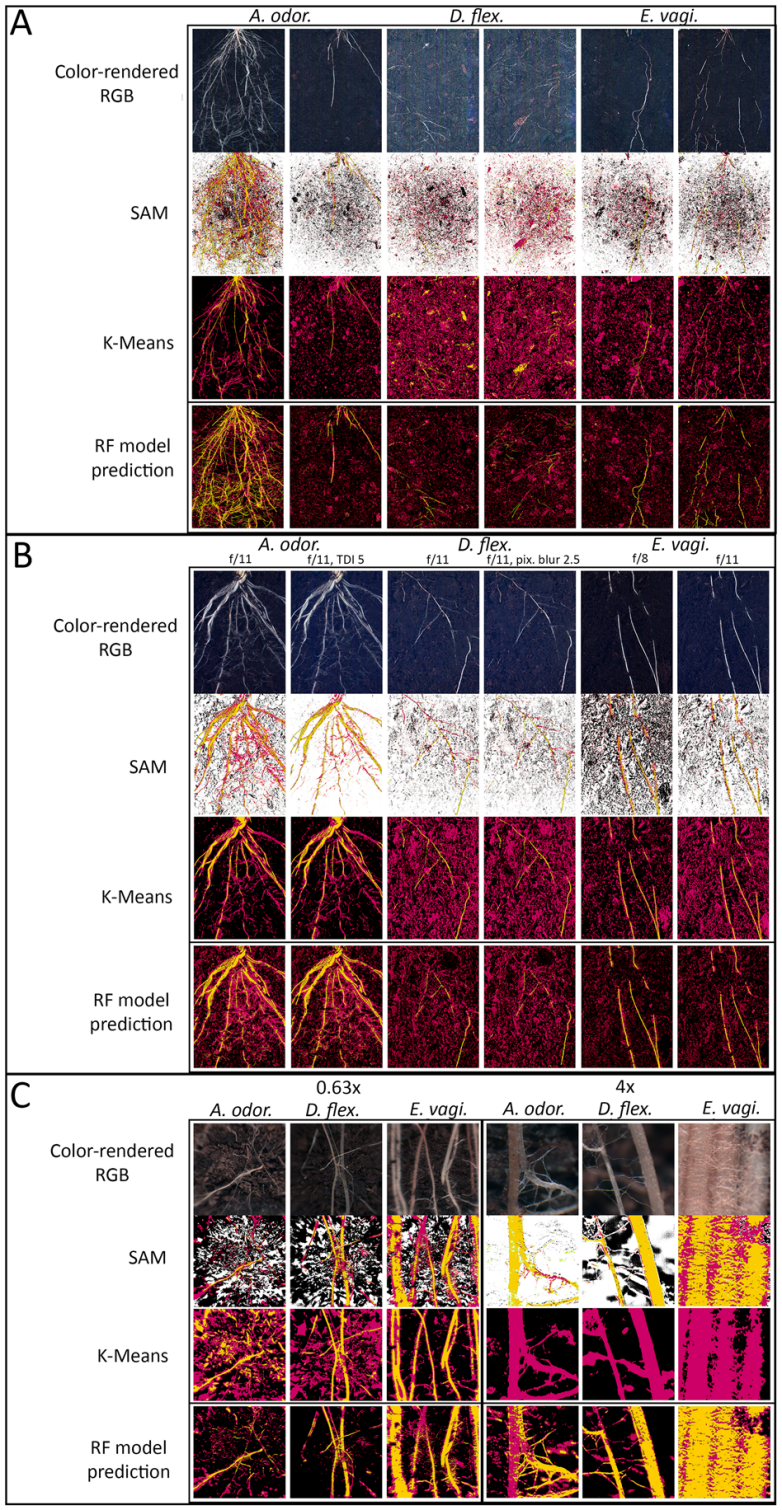
Classification of root systems of three graminoid species in three tested configurations. **A** CONF1 at an aperture of f/5.6 with the following acquisition settings: TDI 3, Pixel blur 2.5, Binning 2 × 2. Two biological replicates for each species were employed. **B** CONF2 with the acquisition settings of TDI 1, Pixel blur 0, Binning 1 × 1, unless otherwise indicated. One biological replicate for each species was imaged under the different acquisition settings. **C** CONF3 with the following acquisition settings: TDI 1, pixel blur 0, binning 1 × 1. One biological replicate for each species was imaged at the different magnifications. In all configurations, the RF model was trained using SAM classifications. *A. odor.* = *A. odoratum, D. flex.* = *D. flexuosa, E. vagi.* = *E. vaginatum*

The differences in root architecture between the three species, as well as the different configuration (CONF) and acquisition settings chosen, had a clear impact on classification performance. For CONF1, which gave the broadest view of the entire root system, the roots of *A. odoratum* and *E. vaginatum* were well-established and classified with great accuracy, while the root system of *D. flexuosa* seemed less vigorous and was not as accurately classified (Fig. 3A). In addition, images for all three species taken with larger apertures (f/2.8 and f/4) suffered from overexposure and poor classification by both SAM classifications and K-Means clustering, which skewed the resulting spectra (Supplementary Fig. 1). After removal of the poor spectral data, only twelve images from an aperture of f/5.6 were used for further data processing.

For CONF2 that was designed with the rhizoboxes positioned closer to the lens, the SAM classifications exhibited slight differences in pixels assigned to the specific classes with different acquisition settings, while there were no discernible differences with K-Means clustering. With SAM classifications, a time delay integration (TDI) of 5 classified fewer soil pixels compared to the standard conditions (TDI of 1), and an aperture of f/8 classified more soil pixels than an aperture of f/11 (Fig. 3B). The difference between a pixel blur of 0 or 2.5 did not lead to any significant changes.

For CONF3 that was tailored to capture magnified sections of various regions within each root system, the accuracy of classifications became less discernible at higher magnifications (Fig. 3C). Specifically, K-Means clustering was only able to distinguish between two classes at a magnification of 1.6x and 4x for most images (images for 1.6x not shown due to similarity with a magnification of 4x). This was due to the presence of much more fine details and specific variation between different root sections. For the few images where the root class was detected, the resulting spectral signatures were incoherent (Supplementary Fig. 2). Therefore, these images were removed from further processing for the K-Means data. In contrast, SAM classification was able to distinguish all three spectral classes for all three tested magnifications (Fig. 3C). The images used for each of the classification methods and further data processing are listed in Table 1.

### Utilizing the second derivative for spectral variable selection enables effective data reduction for machine learning

**Fig. 4:**
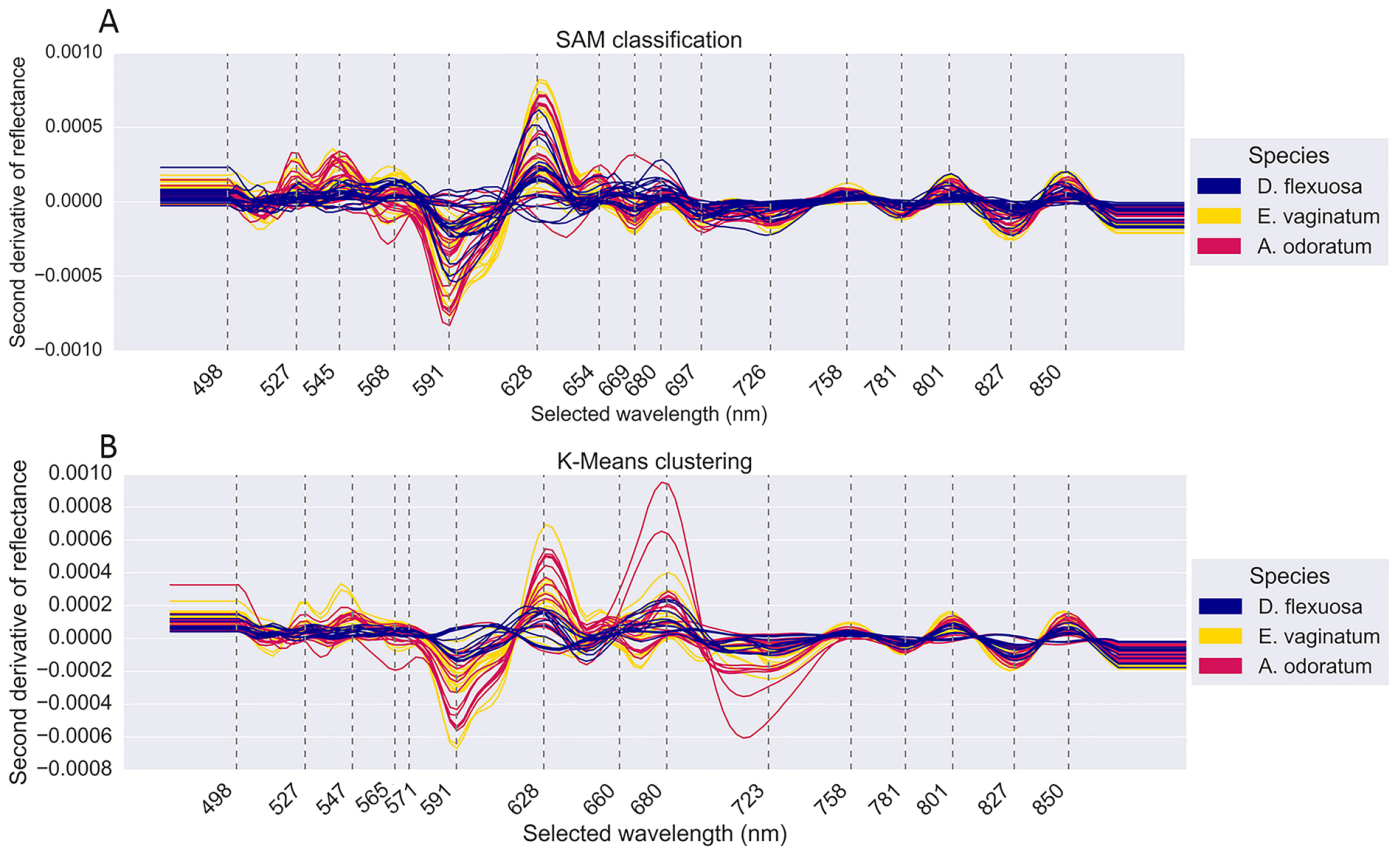
Wavelength selection by Savitzky-Golay smoothing. The second derivatives of the root spectra for all images that were processed in the three configurations of all three graminoid species are shown for (**A**) Spectral Angle Mapper (SAM) classifications and (**B**) K-Means clustering. The wavelengths were selected from the peaks and troughs using the average of each dataset

To identify relevant spectral features and decrease the data size for executing machine learning algorithms, the root spectra was selected to identify the most informative bands. When evaluating the optimal window size for Savitzky-Golay (SG) smoothing using the second derivative, a window of 21 wavelengths proved effective in filtering out noise, while preserving the integrity of signal bands. Smaller windows tended to retain artifact signals, while larger windows exceeding 25 wavelengths may have over-smoothed genuine sample signals (Supplementary Fig. 3). This parameter allowed the data to be reduced from 150 to 16 bands for SAM (Fig. 4A) and 15 bands for K-Means spectra (Fig. 4B), to facilitate expedited processing of machine learning algorithms. The selected wavelengths between the two classification methods slightly differed in the 545 nm—726 nm range.

### Random Forest (RF) models trained on SAM classifications achieve higher accuracy compared to training on K-Means labels

Of the two common machine learning algorithms tested, Random Forest classifier (RF) and Support Vector Machine (SVM), the SVM model took more computational time with similar results to the RF model, and thus the RF model was further used to develop a robust classification model for root systems. For each configuration, two RF models were developed: one trained on the SAM classifications and the other trained on the K-Means classifications, with equal representation of all three graminoid species in the training dataset. One parameter within each configuration was selected to train the models. The chosen parameters were an aperture of f/5.6 for CONF1, an aperture of f/11 for CONF2, and a magnification of 0.63 x for CONF3. Cropping the datacubes and associated classified images to a 300 × 300 pixel area encompassing all three classes, and reducing the datasets to the selected wavelengths (Fig. 4), facilitated a more efficient model training process. Additionally, unlabeled pixels in the datasets classified using SAM were removed. The results from testing the models, indicated that the RF models for the SAM classified images had an 88–91% accuracy (macro average of all per-class F1-scores), while for the K-Means classified images the accuracy was 77–85% (Table 2), which resulted in more pixels being inaccurately predicted to be interface or soil with the K-Means RF model (Supplementary Fig. 4). Therefore, the SAM trained RF model was considered to be superior to the K-Means RF model and was used to predict all other images in each configuration.

**Table 2:** Classification accuracy for Random Forest (RF) models trained on Spectral Angle Mapper (SAM) classifications and K-Means classifications

CONF	Classification	Feature^a^	1. Soil	2. Interface	3. Root	Macro avg
CONF1 (aperture: f/5.6)	SAM	Precision	0.89	0.86	0.94	0.9
		Recall	0.97	0.73	0.88	0.86
		F1-score^b^	**0.93**	**0.79**	**0.91**	**0.88**
		Support	27307	12871	5608	45786
	K-Means	Precision	0.85	0.75	0.89	0.83
		Recall	0.85	0.79	0.56	0.73
		F1-score^b^	**0.85**	**0.77**	**0.69**	**0.77**
		Support	58132	42645	7223	108000
CONF2 (aperture: f/11)	SAM	Precision	0.99	0.89	0.86	0.91
		Recall	0.99	0.9	0.83	0.91
		F1-score^b^	**0.99**	**0.89**	**0.85**	**0.91**
		Support	19233	18884	12,074	50,191
	K-means	Precision	0.91	0.76	0.92	0.86
		Recall	0.86	0.85	0.8	0.84
		F1-score^b^	**0.88**	**0.81**	**0.86**	**0.85**
		Support	90133	59002	12865	162000
CONF3 (magnification: 0.63x)	SAM	Precision	0.95	0.83	0.93	0.90
		Recall	0.96	0.81	0.92	0.90
		F1-score^b^	**0.96**	**0.82**	**0.92**	**0.90**
		Support	74209	30945	30065	135219
	K-means	Precision	0.86	0.71	0.9	0.82
		Recall	0.83	0.77	0.83	0.81
		F1-score^b^	**0.84**	**0.74**	**0.86**	**0.81**
		Support	68874	56367	36759	162000

^a^The precision, recall, F1-scores, and support (number of actual pixels) for each of the three classes (Soil, Interface, Root), and the macro average (arithmetic mean of all per-class F1-scores) for each of the testing datasets for the RF models is provided for each configuration (CONF)

^b^The F1-score, which is a harmonic mean of the precision and recall values, is used as the main metric of accuracy on a scale of 0 to 1, where the closer it is to 1 represents a precise and accurate model

### Model accuracy varies between imaging configurations and data acquisition parameters

**Fig. 5:**
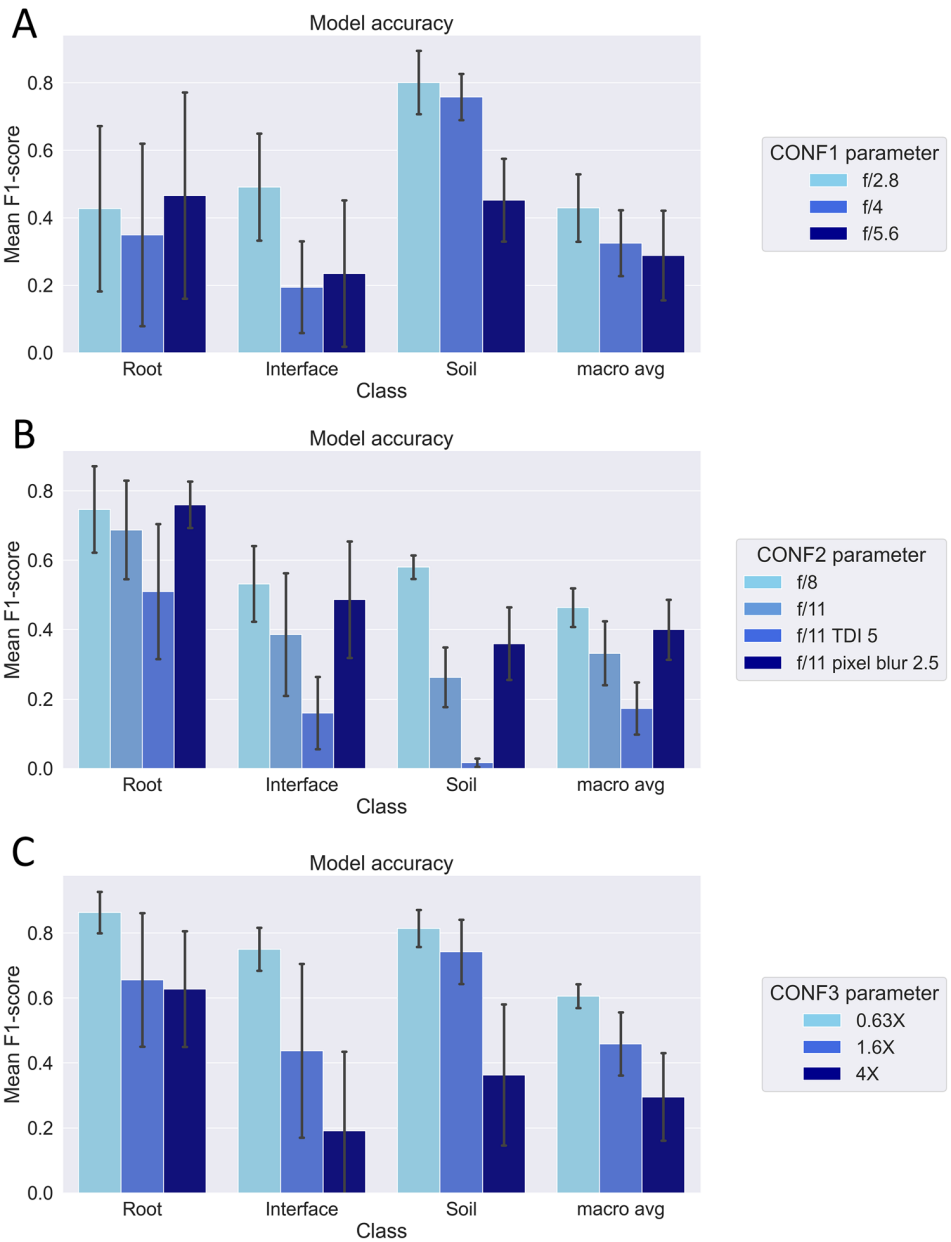
RF model prediction accuracy. The prediction accuracy for each individual class and the macro average accuracy of the combined classes for each acquisition parameter tested within each configuration: **A** CONF1, **B** CONF2, and **C** CONF3. The accuracy is represented by the average ± standard deviation of the F1-scores are as follows: for CONF1, f/2.8 (N = 18), f/4 (N = 18), f/5.6 (N = 30); for CONF2, f/8 (N = 8), f/11 (N = 16), f/11 TDI 5 (N = 3), f/11 pixel blur 2.5 (N = 3); and for CONF3, each magnification (N = 9)

To evaluate the effects of settings selected during image acquisition, the following parameters were methodically adjusted in each configuration: apertures in CONF1; apertures, TDI, and pixel blur settings in CONF2; and magnification lenses in CONF3. Only the accuracy scores for each of the three classes were analyzed since inclusion of unlabelled pixels in the original classified images skewed the calculation for the overall accuracy (macro average). The prediction accuracy in each class as mean F1-scores was found to vary across different configurations and acquisition parameters (Fig. 5).

For CONF1 (Fig. 5A), the root class exhibited the highest variability and lowest accuracy compared to the other configurations (Fig. 5B and C). For CONF2 (Fig. 5B), images taken at an aperture of f/11 with a pixel blur of 2.5 along with an aperture of f/8 showed higher accuracy for all three classes than the images taken at an aperture of f/11 with the standard settings. Since the latter were the images on which the model was trained, this finding was noteworthy. Additionally, the images taken at an aperture of f/11 with a TDI of 5 had the lowest accuracy. For CONF3 (Fig. 5C), a magnification of 0.63x achieved the highest accuracy for all three classes, whereas higher magnifications had lower accuracies. This might be attributed to the fact that the model was trained on the dataset it performed on best. However, training the model using data from all three magnfications led to inaccuracies (Supplementary Fig. 5) and was therefore not feasible. This suggests that each magnification in CONF3 may require a distinct, customized classification model for effective data analysis.

Generally, the accuracy scores during prediction were lower than the testing scores obtained during model training for all configurations. For instance, the root class in CONF2 had a testing accuracy of 85% but a predicted accuracy of only 69% ± 15% for the same aperture on which the model was trained. Similarly, for CONF3 the testing accuracy was 92%, but the predicted accuracy was 86% ± 7% on the same images. Despite these variations, the RF models outperformed either of the previous classification methods for all images (Fig. 3). Due to this, the quality of the spectral signatures for each image needed to be compared to identify the differences between configurations, acquisition parameters, and species.

### Different imaging configurations have the largest impact on spectral signatures

**Fig. 6:**
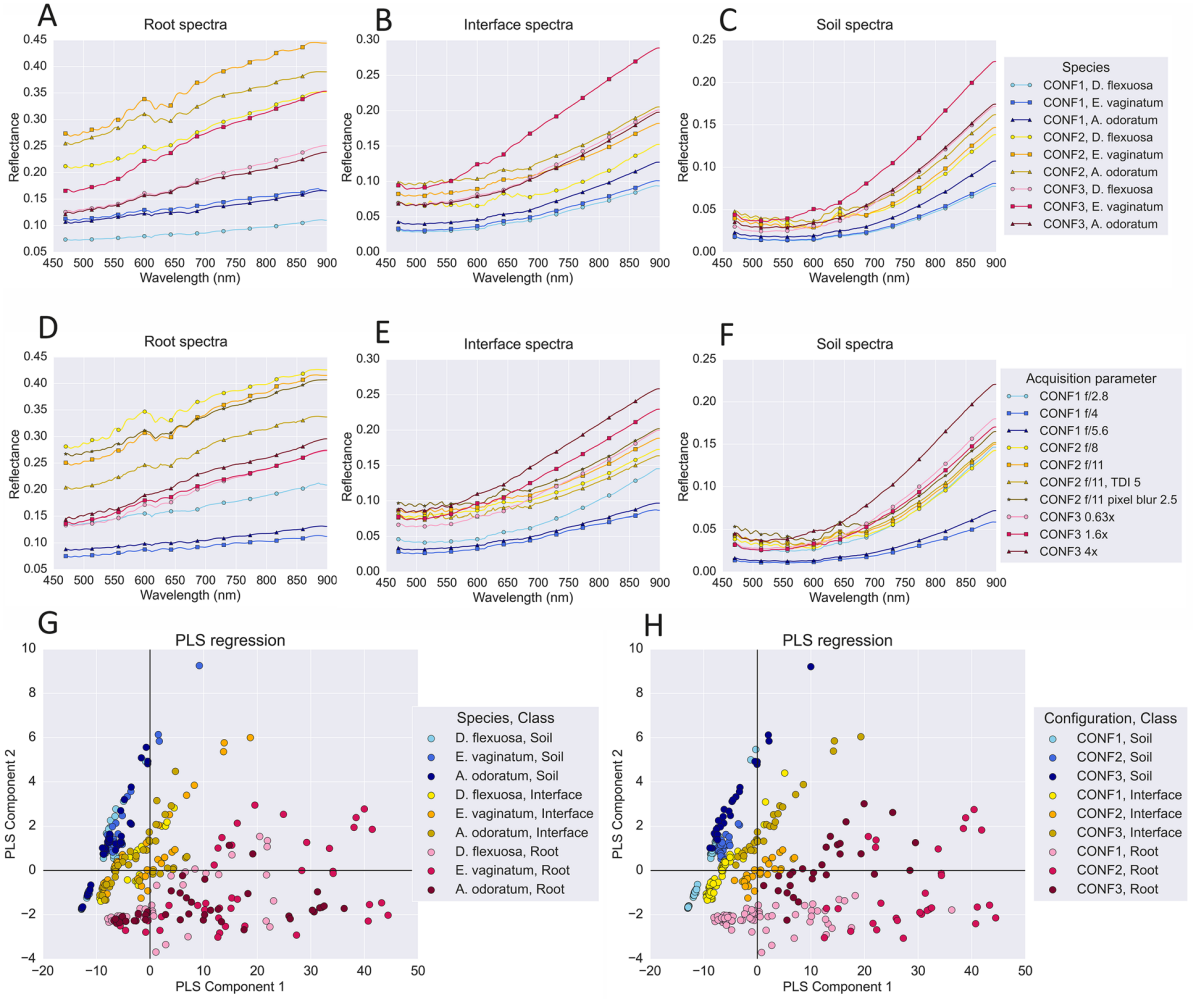
Spectral signatures and Partial Least Squares (PLS) regression of all RF model predicted spectra. The average reflectance spectra across all wavelengths in each class for (**A**-**C**) each species and configuration (CONF) and (**D**-**F**) each acquisition parameter and configuration. The predicted spectra for all data were fitted to (**G**) the combination of each species and class, and (H) the combination of each configuration and class

Spectral analysis showed that the different configurations had the largest effect on spectral signatures, while the different graminoid species had differences in intensity across all three classes within each configuration (Fig. 6A–C). CONF1 and CONF3 produced smoother spectral signatures than those in CONF2, and CONF1 had the lowest intensity of all three configurations. *E. vaginatum* had the highest intensity in all three configurations for the root class, while *D. flexuosa* had the lowest intensity, though it’s spectra was similar to *A. odoratum* in CONF3. *E. vaginatum* had the highest intensity for all three classes in CONF3. CONF2 produced the most variation in spectral signatures within the 550–700 nm range for all three classes compared to CONF1 and CONF3, and the spectral signatures between the three species were similar, only varying in intensity.

The different parameters within each configuration also influenced the spectral signatures (Fig. 6D–F). Spectral signature characteristics remained consistent between apertures f/8.0 and f/11.0 in CONF2, only varying in intensity. A TDI of 5 at an aperture of f/11 in CONF2 resulted in smoother signatures with lower intensity for the root class. In CONF3, a magnification of 4x had the highest intensity for all three classes, but a smoother spectral signature. A Partial Least Squares (PLS) regression confirmed that the three classes correlated most strongly, irrespective of species (Fig. 6G), however, the three configurations also correlated within each class (Fig. 6H).

### Estimation of root biomass with HSI

**Fig. 7:**
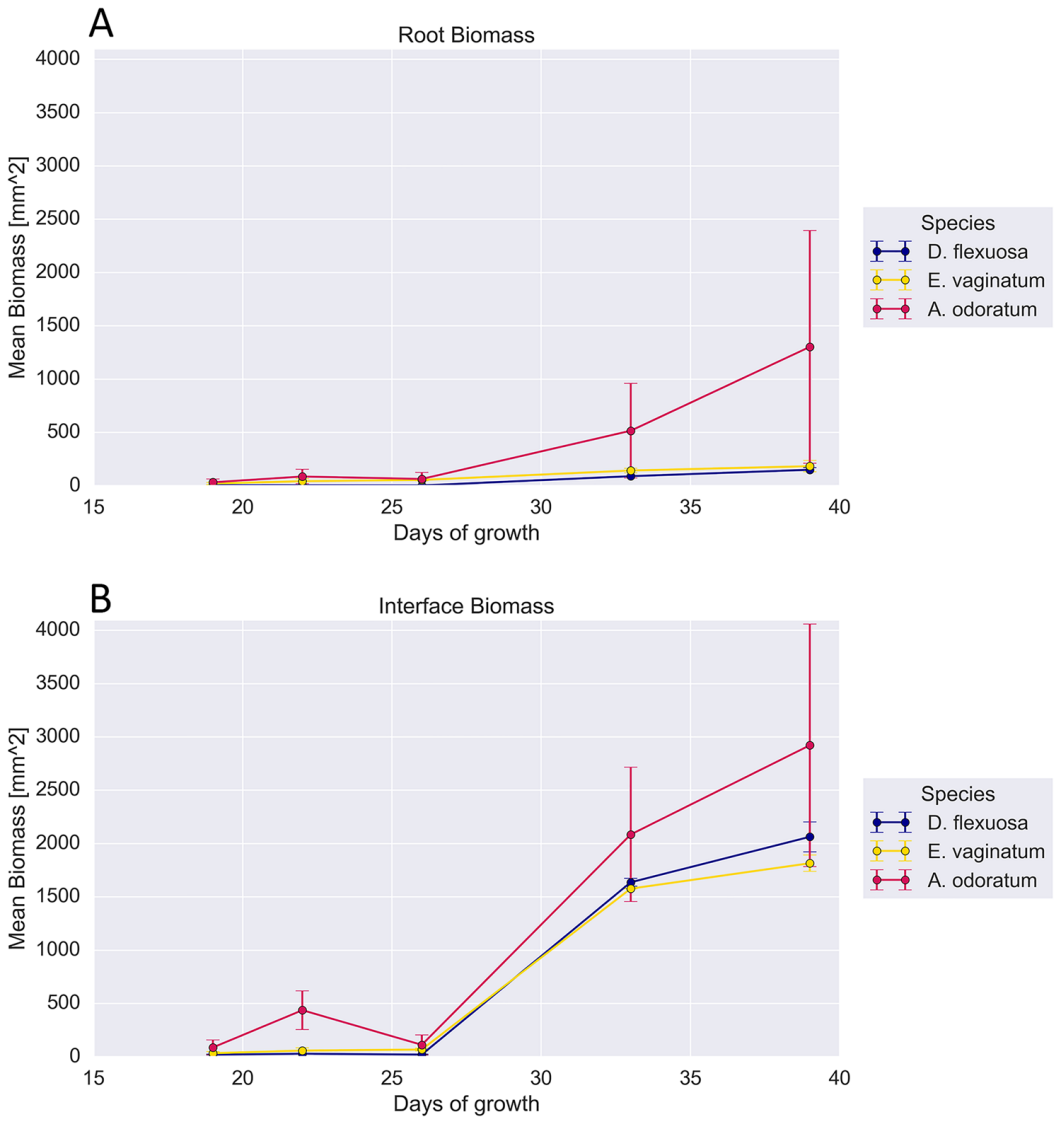
Estimated surface biomass for the root and interface classes. The estimated biomass for the (**A**) root, and (**B**) interface classes in CONF1 over the 20-day imaging period. Biomass was calculated from the area of the image and percentage of pixels in each class. Each point is the average ± standard error of two biological replicates

Since CONF1 provided a full overview of the root systems, it was used to estimate root biomass alongside their spectral signature characterization by HSI. Over the observed period (20 days), the root systems of *A. odoratum* had the highest increase in estimated root biomass over the time course (Fig. 7A), however there was substaintial variance between the two biological replicates. The estimated biomass for the interface classes showed a greater increase than that of the root class (Fig. 7B).

## Discussion

To evaluate the strengths, weaknesses, potentials, and pitfalls of HSI technology in root biological applications, we used the imec VNIR SNAPSCAN camera to image rhizobox-grown root systems of three anatomically different graminoid species. This camera, with its high spatial resolution of up to 3650 × 2048 pixels, was chosen for its ability to capture detailed images compared to other NIR or MWIR HSI cameras that utilize linescan technology. Although these cameras may offer wider spectral resolutions, their spatial resolutions are typically more limited [40]. Additionally, the ability to mount the imec camera on a microscope further enhances its capability to distinguish between the root system and surrounding soil. Image segmentation and classification is a vital aspect of HSI analysis, which have proven to be challenging for agricultural tasks such as detecting disease and pest damage on leaves [41, 42], or identifying weeds in crops fields [43]. Segmenting roots from soil presents even greater challenges due the heterogenous nature of soil and, depending on the soil type, the potential spectral similarities between dry soil and living roots or wet soil and dead roots [18, 44]. Thus, high resolution hyperspectral images are necessary to accurately classify soil-grown root systems.

In our study, we focused on distinguishing roots and soil as the two most obvious ROIs. In addition, we attempted to classify the root-soil interface/rhizosheath where root exudates change the physical and physiological characteristics of the soil, which is a region of high biological and biotechnological interest [45, 46]. However, due to the complex nature of the rhizosheath, this ROI was not well resolved, either spatially or spectrally, and included varying proportions of root and soil areas across all images. To our knowledge, there have been no previous attempts to use HSI for detecting the rhizosheath. However, there is evidence suggesting that the presence of fungi, mold, algae, and differences in soil water potential can be classified to provide insights into the interactions between roots and the rhizosphere [17, 18]. Despite its challenges, the interface ROI was kept as it helped to increase the distinction between the root and soil classes. Utilizing three classes throughout our analyses enabled a comparative analysis of spectral signatures across different configurations, acquisition parameters, and species.

### Imaging configuration and acquisition parameters influence the reflectance spectra

For optimal data capture, selecting the appropriate imaging configuration and acquisition settings tailored to the specific application is crucial. In spectral cameras equipped with an integrated thin-film Fabry–Perot filter, such as the imec SNAPSCAN camera, the position of each filter on the sensor and the aperture size can shift the measured spectra, potentially resulting in a loss of detail, particularly with larger apertures [47]. This may be the reason for the loss of spectral detail in CONF1 in comparison to CONF2, however, the smoother signatures obtained for CONF1 could also be attributted to the images being binned (2 × 2) and having a Time Delay Integration (TDI) of 3. Pixel binning can be set from 1 to 20 to merge pixels in a NxN neighborhood, where the benefits of binning can increase the signal to noise ratio (SNR), which is optimal for larger ROIs to reduce individual pixel noise [48], but will inevitably decrease the spatial resolution. TDI sets the step size (1–5) for imaging each spectral band, where lower values allow information for pixels in the same spectral band to be averaged across multiple frames, thus significantly improving the SNR [49]. The effect of no TDI (a setting of 5) was seen in CONF2 which resulted in smoother spectral signatures with lower intensity. In CONF3, using the highest magnification factor (4x) led to a loss of detail within the spectral signature. This outcome could be expected, considering that the spectral data from other configurations represented averages across larger regions of the root system, while at 4x magnification, the spectral signature was derived from just 0.25 cm^2^ sections of the root system. The variation in spectral signatures across different acquisition settings highlights the necessity of maintaining consistent conditions throughout a study to ensure comparable results.

### Different imaging configurations can be utilized for various biological inquiries

The three configurations used in this study are adaptable for various biological questions, with the choice of acquisition parameters being contingent on the specific application. In CONF1, where the sample is positioned at the greatest distance from the lens, an overview of the root system architecture is achievable, which can be utilized to track general changes in composition and biomass allocation over time. However, the spectral signatures derived from the acquisition parameters used here should be viewed with skepticism. The binning, TDI, and lens aperture settings may not be optimal for this configuration and should be further investigated. Additionally, biomass estimates from these images should be viewed as approximations since they only capture the visible surface portion and exclude the biomass hidden within the 0.6 cm depth of the rhizoboxes. Since the interface class generally consisted of a heterogenous mix of fine roots, root hairs, and soil, interpreting its biomass in a biological context here is not feasible. Nevertheless, it highlights that these parts of the root systems contribute to the overall root biomass. Given the crucial role these regions play in rhizosheath formation and their complex interactions with the surrounding soil [45, 50], this class holds potential for further investigation into root-soil interface dynamics. Furthermore, the correlation between the estimated surface-level root biomass from classification outcomes and the actual biomass was not explored or confirmed in this study. This point is shown to demonstrate the method's potential applicability in future research, particularly in studies focused on the tradeoffs between root trait plasticity and belowground biomass allocation as key indicators of plant resilience to environmental stresses [51].

In CONF2, positioning the sample closer to the lens allows for a more detailed analysis of specific root system segments with clear distinctions in spectral signatures. This proximity enhances resolution and detail, which is essential for linking HSI data with specific root functional traits and compositional variations. The differences in spectral signatures between the root and soil classes were similar to other studies, where root signatures had higher average reflectance and greater variance than the surrounding soil [18]. When combined with deep-learning models, this configuration is optimal for classifying chemical or nutrient concentrations and detecting related stresses or resilience in root systems [15]. Although this study primarily focused on comparing imaging configurations and acquisition settings, the insights gained lay a foundation for future research. These studies could explore the relationships between root traits and environmental adaptations, thereby deepening our understanding of root strategies and their ecological impacts during environmental changes [5, 52].

Future research should focus on refining the set-up and data analysis methods for CONF3 using the stereomicroscope. Although distinguishing the root system from the soil was achievable, accurately capturing the intricate details of smaller root segments might require a more extensive classification system. With adjustments to the imaging set-up and analytical approach, this configuration holds potential for detailed investigations of fine-scale root-soil interactions and the role of root exudates in the rhizosphere [17, 52]. Furthermore, adapting this set-up for use with a brightfield microscope could yield significant insights into root physiological processes, a topic that has not yet been thoroughly explored in the realm of HSI. The purpose of employing CONF3 in this study was to demonstrate its capabilities and to benchmark it against broader-scale configurations.

### Choosing image-processing techniques depends on the objective for hyperspectral data analysis

When processing hyperspectral images, a variety of analytical techniques are available, and selecting the appropriate steps depends on both the priority of the data outcome and the availability of methods. Since our goal was to assess both the ability to distinguish root systems from the soil matrix, as well as the influence of configuration and acquisition parameters on data quality, a data-processing pipeline was developed based on common techniques to reduce bias, enhance reproducibility, and increase efficiency. Utilizing the supervised Spectral Angle Mapper (SAM) classification from the SNAPSCAN software, which groups pixels by spectral angle similarity to the reference vector [53], alongside the widely used unsupervised classification method, K-Means clustering [54], allowed for a quick assessment of root classification accuracy. In general, both classification methods exhibited advantages and limitations. The supervised SAM classification method captured very fine details of the root system; however, it fell short in classifying all pixels in the image. On the other hand, unsupervised K-Means clustering was more automated than SAM, classifying each image into three classes with a single line of code, however the detailed root regions identified with SAM were lost and often classified as the interface class. Despite K-Means being one of the most widely used clustering algorithms, it has been found to often perform poorly [55], and this seemed to be the case here. Although the K-Means algorithm can be enhanced with adaptive initialization methods [55], and more sophisticated clustering algorithms such as Artificial Neural Networks (ANN) might surpass SAM in performance [56], these approaches typically demand greater computational resources. These options were not explored in this study as they were beyond the scope of the research. Ultimately, classification accuracy depends on various factors, necessitating further processing steps for de-noising, data reduction, and image compression to extract the relevant information [28].

Data reduction is a necessary step for handling the large data size of hyperspectral images to reduce computational load and time. This can be done through spatial or spectral binning, or various variable selection approaches [57]. The SNAPSCAN software offers spatial binning capabilities but given the significance of the spatial variations between the root system and the soil at the pixel scale, this approach did not appear to be the best method for enhancing spectral distinctions. Calculating the second derivative using Savitzky-Golay (SG) smoothing is a widely used spectral pre-processing technique that enhances desired spectral features while reducing unwanted noise from the sample or instrument, such as refractive index scattering and white noise [36]. The primary objective of the image processing was to distinguish root systems from the surrounding soil, so the second derivative was applied only to the root spectra. Since there is no general method for selecting the optimal window size for SG-smoothing [36], testing a range of window sizes enabled the identification of consistent signals across multiple ranges. These signals likely reflect wavelengths where there are true sample differences and assist in reducing artifact noise in the spectra. Using these wavelengths as the method for variable selection facilitated a significant reduction in data, from 150 to 15 wavelengths. This enabled the effective application of machine learning to evaluate the accuracy of models in predicting root, soil, and interface regions within images. Alternatively, Principal Component Analysis (PCA) could have been a suitable method to reduce the dimensionality of the data. However, the optimal wavelengths between PCA loadings and second derivative spectra have shown similarity between different sample sets [58], therefore variable selection by the second derivative was an effective choice for data reduction.

### Machine learning-based hyperspectral image classification provides a robust method to study root systems

Both machine learning algorithms initially tested in this study, Support Vector Machine (SVM) and Random Forest (RF) classification, are commonly used classifiers [59, 60]. However, due to faster computational time, RF was the preferred algorithm to generate the classification models in this study. Consistent with the initial classification results, the SAM method produced higher accuracy scores when generating the models. Visually, the RF model demonstrated exceptional accuracy in predicting root and soil regions and outperformed the initial SAM classifications in all configurations. However, the accuracy scores did not reflect this assessment. During model testing, the accuracy for the root class ranged from 85–92%, but it dropped to 35–86% when predicting the original images. Beyond the differences in acquisition settings, the low F1-scores observed in the predicted images can likely be attributed to the model being trained on selectively cropped regions, which contained the most accurately classified sections of an image. In contrast, predictions were performed on entire images, which displayed variations in classification quality. This discrepancy was particularly noticeable in CONF3, where the stereomicroscope's lens limitations resulted in image vignetting. Furthermore, the deviation in accuracy scores within each parameter suggests variability in accuracy over time and across the three species examined. The low accuracy scores might also be explained by the evaluation metric of the RF models' accuracy, which used the assumption that the original SAM labels were accurate. However, these labels may not accurately represent the true classification of each pixel. The SAM classifications were derived from subjective choices in the manual selection of ROIs that constitute only a small fraction of the total pixels. The classification of the root-soil interface class was often confounded by variations in the background soil or root systems, rather than the interface itself. Adding more classes to differentiate these regions may have been useful but can also lead to increased subjective bias. Although numerous other model classifiers and image processing methods hold the potential of delivering more precise segmentation of root system regions [12, 18, 61], their exploration was beyond the scope of this particular study. Analyzing the spectral signatures for each class was an effective approach to determine how various configurations and acquisition settings impacted data quality. Future research should build upon our findings by investigating other methods tailored to the specific goals of the analysis.

## Conclusions

In this study, we evaluated various HSI acquisition parameters and data processing techniques for analyzing root systems across three different imaging scales using the VNIR SNAPSCAN HSI camera. Our methodology, which involved use of supervised Spectral Angle Mapper (SAM) for initial image classification into roots, soil, and the root-soil interface, followed by selection of variables through the use of second derivative and training a Random Forest (RF) model, provided a robust framework for image classification. This approach effectively highlighted the spectral signature differences across the three configurations and acquisition parameters. The analysis method could be successfully used for all three graminoid species tested, despite differences in their root architecture. The scripts developed during this study are available as an online Python-based tool for semi-automated HSI analysis, offering a scalable framework that can be expanded and refined with further techniques.

## Supplementary Information

Additional file 1: Supplementary Figure 1. The effect of different apertures on the selection spectra from SAM classifications and K-Means clustering for all data from CONF1. Supplementary Figure 2. The resulting spectra from K-Means clustering in CONF3 at magnification factors of 1.6x and 4x. Supplementary Figure 3. Window size comparison for the second derivative with Savitzy-Golay smoothed root spectra. Supplementary Figure 4. Confusion matrix for random forest models trained on Spectral Angle mapper classifications or K-Means classifications. Supplementary Figure 5. Model predicted image of *A. odoratum *at 0.63x magnification when the model was trained on all magnification factors.

## Data Availability

The scripts for data analysis are available from https://github.com/corinef/Automated-root-classification.

## References

[1] Ndour PMS, Heulin T, Achouak W, Laplaze L, Cournac L. The rhizosheath: from desert plants adaptation to crop breeding. Plant Soil. 2020;456:1–13. 10.1007/s11104-020-04700-3.

[2] Lynch JP. Roots of the second green revolution. Aust J Bot. 2007;55(5):493–512. 10.1071/BT06118.

[3] Feeney DS, Crawford JW, Daniell T, Hallett PD, Nunan N, Ritz K, Rivers M, Young IM. Three-dimensional microorganization of the soil–root–microbe system. Microb Ecol. 2006;52:151–8. 10.1007/s00248-006-9062-8. 16680511 10.1007/s00248-006-9062-8

[4] Nwokolo NL, Enebe MC, Chigor CB, Chigor VN, Dada OA. The contributions of biotic lines of defence to improving plant disease suppression in soils: a review. Rhizosphere. 2021;19: 100372. 10.1016/j.rhisph.2021.100372.

[5] Bodner G, Alsalem M, Nakhforoosh A. Root system phenotying of soil-grown plants via RGB and hyperspectral imaging. Methods Mol Biol. 2021;2264:245–68. 10.1007/978-1-0716-1201-9_17. 33263915 10.1007/978-1-0716-1201-9_17

[6] Schmidt JE, Lowry C, Gaudin ACM. An optimized rhizobox protocol to visualize root growth and responsiveness to localized nutrients. J Vis Exp. 2018;140:58674. 10.3791/58674. 10.3791/58674 PMC6235577 30394399

[7] Nagel KA, Putz A, Gilmer F, Heinz K, Fischbach A, Pfeifer J, Faget M, Blossfeld S, Ernst M, Dimaki C, et al. GROWSCREEN-Rhizo is a novel phenotyping robot enabling simultaneous measurements of root and shoot growth for plants grown in soil-filled rhizotrons. Funct Plant Biol. 2012;39(11):891–904. 10.1071/FP12023. 32480839 10.1071/FP12023

[8] Bontpart T, Concha C, Giuffrida MV, Robertson I, Admkie K, Degefu T, Girma N, Tesfaye K, Haileselassie T, Fikre A, et al. Affordable and robust phenotyping framework to analyse root system architecture of soil-grown plants. Plant J. 2020;103(6):2330–43. 10.1111/tpj.14877. 32530068 10.1111/tpj.14877

[9] Rellán-Álvarez R, Lobet G, Lindner H, Pradier P-L, Sebastian J, Yee M-C, Geng Y, Trontin C, LaRue T, Schrager-Lavelle A, et al. GLO-Roots: an imaging platform enabling multidimensional characterization of soil-grown root systems. Elife. 2015;4: e07597. 10.7554/eLife.07597. 26287479 10.7554/eLife.07597 PMC4589753

[10] Rahman G, Sohag H, Chowdhury R, Wahid KA, Dinh A, Arcand M, Vail S. SoilCam: a fully automated minirhizotron using multispectral imaging for root activity monitoring. Sensors. 2020;20(3):787. 10.3390/s20030787. 32023975 10.3390/s20030787 PMC7038518

[11] Handy G, Carter I, Mackenzie AR, Esquivel-Muelbert A, Smith AG, Yaffar D, Childs J, Arnaud M. Variation in forest root image annotation by experts, novices, and AI. Plant Methods. 2024;20(1):154. 10.1186/s13007-024-01279-z. 39350215 10.1186/s13007-024-01279-z PMC11443924

[12] Bodner G, Nakhforoosh A, Arnold T, Leitner D. Hyperspectral imaging: a novel approach for plant root phenotyping. Plant Methods. 2018;14:84. 10.1186/s13007-018-0352-1. 30305838 10.1186/s13007-018-0352-1 PMC6169016

[13] Park E, Kim Y-S, Faqeerzada MA, Kim MS, Baek I, Cho B-K. Hyperspectral reflectance imaging for nondestructive evaluation of root rot in Korean ginseng (*Panax**ginseng* Meyer). Front Plant Sci. 2023. 10.3389/fpls.2023.1109060. 36818876 10.3389/fpls.2023.1109060 PMC9930644

[14] Shao Y, Ji S, Xuan G, Ren Y, Feng W, Jia H, Wang Q, He S. Detection and analysis of chili pepper root rot by hyperspectral imaging technology. Agronomy. 2024;14(1):226. 10.3390/agronomy14010226.

[15] Zhou X, Zhao C, Sun J, Yao K, Xu M. Detection of lead content in oilseed rape leaves and roots based on deep transfer learning and hyperspectral imaging technology. Spectrochim Acta Part A Mol Biomol Spectrosc. 2023;290: 122288. 10.1016/j.saa.2022.122288. 10.1016/j.saa.2022.122288 36608517

[16] Xu Z, Hu H, Wang T, Zhao Y, Zhou C, Xu H, Mao X. Identification of growth years of Kudzu root by hyperspectral imaging combined with spectral–spatial feature tokenization transformer. Comput Electron Agric. 2023;214: 108332. 10.1016/j.compag.2023.108332.

[17] Nakaji T, Noguchi K, Oguma H. Classification of rhizosphere components using visible–near infrared spectral images. Plant Soil. 2008;310:245–61. 10.1007/s11104-007-9478-z.

[18] Chang SJ, Chowdhry R, Song Y, Mejia T, Hampton A, Kucharski S, Sazzad TM, Zhang Y, Koppal SJ, Wilson CH, et al. HyperPRI: a dataset of hyperspectral images for underground plant root study. Comput Electron Agric. 2024;225: 109307. 10.1016/j.compag.2024.109307.

[19] Song Y, Sapes G, Chang S, Chowdhry R, Mejia T, Hampton A, Kucharski S, Sazzad TMS, Zhang Y, Tillman BL, et al. Hyperspectral signals in the soil: plant–soil hydraulic connection and disequilibrium as mechanisms of drought tolerance and rapid recovery. Plant, Cell Environ. 2024;47(11):4171–87. 10.1111/pce.15011. 38924477 10.1111/pce.15011

[20] Großkinsky DK, Svensgaard J, Christensen S, Roitsch T. Plant phenomics and the need for physiological phenotyping across scales to narrow the genotype-to-phenotype knowledge gap. J Exp Bot. 2015;66(18):5429–40. 10.1093/jxb/erv345. 26163702 10.1093/jxb/erv345

[21] Jammer A, Akhtar SS, Amby DB, Pandey C, Mekureyaw MF, Bak F, Roth PM, Roitsch T. Enzyme activity profiling for physiological phenotyping within functional phenomics: plant growth and stress responses. J Exp Bot. 2022;73(15):5170–98. 10.1093/jxb/erac215. 35675172 10.1093/jxb/erac215

[22] Liu K-H, Yang M-H, Huang S-T, Lin C. Plant species classification based on hyperspectral imaging via a lightweight convolutional neural network model. Front Plant Sci. 2022;13: 855660. 10.3389/fpls.2022.855660. 35498669 10.3389/fpls.2022.855660 PMC9044035

[23] Garillos-Manliguez CA, Chiang JY. Multimodal deep learning and visible-light and hyperspectral imaging for fruit maturity estimation. Sensors. 2021;21(4):1288. 10.3390/s21041288. 33670232 10.3390/s21041288 PMC7916978

[24] Amziane A, Losson O, Mathon B, Dumenil A, Macaire L. Reflectance estimation from multispectral linescan acquisitions under varying illumination—application to outdoor weed identification. Sensors. 2021;21(11):3601. 10.3390/s21113601. 34064243 10.3390/s21113601 PMC8196826

[25] Brunner A, Schmidt VM, Zelger B, Woess C, Arora R, Zelger P, Huck CW, Pallua J. Visible and near-infrared hyperspectral imaging (HSI) can reliably quantify CD3 and CD45 positive inflammatory cells in myocarditis: pilot study on formalin-fixed paraffin-embedded specimens from myocard obtained during autopsy. Spectrochim Acta Part A Mol Biomol Spectrosc. 2022;274: 121092. 10.1016/j.saa.2022.121092. 10.1016/j.saa.2022.121092 35257987

[26] Kotronias RA, Fielding K, Greenhalgh C, Lee R, Alkhalil M, Marin F, Emfietzoglou M, Banning AP, Vallance C, Channon KM, et al. Machine learning assisted reflectance spectral characterisation of coronary thrombi correlates with microvascular injury in patients with ST-segment elevation acute coronary syndrome. Front Cardiovas Med. 2022;9: 930015. 10.3389/fcvm.2022.930015. 10.3389/fcvm.2022.930015 PMC9530633 36204570

[27] Vandenabeele M, Veys L, Lemmens S, Hadoux X, Gelders G, Masin L, Serneels L, Theunis J, Saito T, Saido TC, et al. The App^NL-G-F^ mouse retina is a site for preclinical Alzheimer’s disease diagnosis and research. Acta Neuropathologica Commun. 2021. 10.1186/s40478-020-01102-5. 10.1186/s40478-020-01102-5 PMC7788955 33407903

[28] Vidal M, Amigo JM. Pre-processing of hyperspectral images. essential steps before image analysis. Chemometrics Intell Lab Syst. 2012;117:138–48. 10.1016/j.chemolab.2012.05.009.

[29] Balsbergpahlsson AM. Growth, radicle and root hair development of *Deschampsia**flexuosa* (L.) Trin. seedlings in relation to soil acidity. Plant Soil. 1995;175:125–32. 10.1007/BF02413017.

[30] Chen W, Tape KD, Euskirchen ES, Liang S, Matos A, Greenberg J, Fraterrigo JM. Impacts of arctic shrubs on root traits and belowground nutrient cycles across a northern Alaskan climate gradient. Front Plant Sci. 2020;11: 588098. 10.3389/fpls.2020.588098. 33362815 10.3389/fpls.2020.588098 PMC7758488

[31] Gould B, McCouch S, Geber M. *De**novo* transcriptome assembly and identification of gene candidates for rapid evolution of soil Al tolerance in *Anthoxanthum**odoratum* at the long-term park grass experiment. PLoS ONE. 2015;10(7): e0124424. 10.1371/journal.pone.0124424. 26148203 10.1371/journal.pone.0124424 PMC4493143

[32] Boggs T, March D, McGibbney LJ, Magimel F, Mason G, Banman K, Jouni M, Kumar R, et al. The Gitter Badger spectralpython/spectral: spectral Python (SPy). 2022. Zenodo. 10.5281/zenodo.7135091.

[33] The Pandas Development Team. Pandas-dev/pandas: Pandas (v203). 2023. Zenodo. 10.5281/zenodo.8092754.

[34] Savitzky A, Golay MJ. Smoothing and differentiation of data by simplified least squares procedures. Anal Chem. 1964;36(8):1627–39. 10.1021/ac60214a047.

[35] Virtanen P, Gommers R, Oliphant TE, Haberland M, Reddy T, Cournapeau D, Burovski E, Peterson P, Weckesser W, Bright J, et al. SciPy 1.0: fundamental algorithms for scientific computing in Python. Nat Methods. 2020;17:261–72. 10.1038/s41592-019-0686-2. 32015543 10.1038/s41592-019-0686-2 PMC7056644

[36] Zimmermann B, Kohler A. Optimizing Savitzky-Golay parameters for improving spectral resolution and quantification in infrared spectroscopy. Appl Spectrosc. 2013;67(8):892–902. 10.1366/12-06723. 23876728 10.1366/12-06723

[37] Clark A. Pillow (PIL Fork) documentation. Readthedocs. 2015. https://buildmedia.readthedocs.org/media/pdf/pillow/latest/pillow.pdf. Accessed 1 Jul 2023.

[38] Pedregosa F, Varoquaux G, Gramfort A, Michel V, Thirion B, Grisel O, Blondel M, Prettenhofer P, Weiss R, Dubourg V, et al. Scikit-learn: machine learning in Python. J Machine Learn Res. 2011;12:2825.

[39] Kleinbaum DG, Kupper LL, Muller KE, Nizam A. Applied regression analysis and multivariable methods. 3rd ed. Pacific Grove, Ca: Brooks/Cole Publishing Company; 1998.

[40] Manley M. Near-infrared spectroscopy and hyperspectral imaging: non-destructive analysis of biological materials. Chem Soc Rev. 2014;43(24):8200–14. 10.1039/C4CS00062E. 25156745 10.1039/c4cs00062e

[41] Krüger M, Zemanek T, Wuttke D, Dinkel M, Serfling A, Böckmann E. Hyperspectral imaging for pest symptom detection in bell pepper. Plant Methods. 2024;20:156. 10.1186/s13007-024-01273-5. 39358772 10.1186/s13007-024-01273-5 PMC11447932

[42] Singh V, Misra AK. Detection of plant leaf diseases using image segmentation and soft computing techniques. Inform Proc Agricu. 2017;4(1):41–9. 10.1016/j.inpa.2016.10.005.

[43] Liu T, Zhao Y, Wang H, Wu W, Yang T, Zhang W, Zhu S, Sun C, Yao Z. Harnessing UAVs and deep learning for accurate grass weed detection in wheat fields: a study on biomass and yield implications. Plant Methods. 2024;20:144. 10.1186/s13007-024-01272-6. 39300566 10.1186/s13007-024-01272-6 PMC11412042

[44] Zhang Y, Sazzad TM, Song Y, Chang SJ, Chowdhry R, Mejia T, Hampton A, Kucharski S, Gerber S, Tillman B, et al. 2024 Cost-efficient active illumination camera for hyperspectral reconstruction*.* arXiv:2406.19560v1. 10.48550/arXiv.2406.19560.

[45] Aslam MM, Karanja JK, Dodd IC, Waseem M, Weifeng X. Rhizosheath: an adaptive root trait to improve plant tolerance to phosphorus and water deficits? Plant, Cell Environ. 2022;45(10):2861–74. 10.1111/pce.14395. 35822342 10.1111/pce.14395 PMC9544408

[46] Galloway AF, Knox P, Krause K. Sticky mucilages and exudates of plants: putative microenvironmental design elements with biotechnological value. New Phytol. 2020;225(4):1461–9. 10.1111/nph.16144. 31454421 10.1111/nph.16144

[47] Goossens T, Geelen B, Pichette J, Lambrechts A, Van Hoof C. Finite aperture correction for spectral cameras with integrated thin-film Fabry-Perot filters. Appl Opt. 2018;57(26):7539–49. 10.1364/AO.57.007539. 30461824 10.1364/AO.57.007539

[48] Riccioli C, Pérez-Marín D, Garrido-Varo A. Optimizing spatial data reduction in hyperspectral imaging for the prediction of quality parameters in intact oranges. Postharvest Biol Technol. 2021;176: 111504. 10.1016/j.postharvbio.2021.111504.

[49] Fan X, Liu C, Liu S, Xie Y, Zheng L, Wang T, Feng Q. The instrument design of lightweight and large field of view high-resolution hyperspectral camera. Sensors. 2021;21(7):2276. 10.3390/s21072276. 33805129 10.3390/s21072276 PMC8037560

[50] Galloway AF, Akhtar J, Marcus SE, Fletcher N, Field K, Knox P. Cereal root exudates contain highly structurally complex polysaccharides with soil-binding properties. Plant J. 2020;103(5):1666–78. 10.1111/tpj.14852. 32463959 10.1111/tpj.14852

[51] Chandregowda MH, Tjoelker MG, Pendall E, Zhang H, Churchill AC, Power SA. Belowground carbon allocation, root trait plasticity, and productivity during drought and warming in a pasture grass. J Exp Bot. 2023;74(6):2127–45. 10.1093/jxb/erad021. 36640126 10.1093/jxb/erad021 PMC10084810

[52] Williams A, Langridge H, Straathof AL, Muhamadali H, Hollywood KA, Goodacre R, de Vries FT. Root functional traits explain root exudation rate and composition across a range of grassland species. J Ecol. 2022;110:21–33. 10.1111/1365-2745.13630.

[53] Kruse FA, Lefkoff AB, Boardman JW, Heidebrecht KB, Shapiro AT, Barloon PJ, Goetz AFH. The spectral image processing system (SIPS)—interactive visualization and analysis of imaging spectrometer data. Remote Sens Environ. 1993;44(2–3):145–63. 10.1016/0034-4257(93)90013-N.

[54] Sinaga KP, Yang MS. Unsupervised K-Means clustering algorithm. IEEE Access. 2020;8:80716–27. 10.1109/ACCESS.2020.2988796.

[55] Celebi ME, Kingravi HA, Vela PA. A comparative study of efficient initialization methods for the K-Means clustering algorithm. Expert Syst Appl. 2013;40:200–10. 10.1016/j.eswa.2012.07.021.

[56] Petropoulos GP, Vadrevu KP, Xanthopoulos G, Karantounias G, Scholze M. A comparison of spectral angle mapper and artificial neural network classifiers combined with landsat TM imagery analysis for obtaining burnt area mapping. Sensors. 2010;10(3):1967–85. 10.3390/s100301967. 22294909 10.3390/s100301967 PMC3264462

[57] De Juan A, Piqueras S, Maeder M, Hancewicz T, Duponchel L, Tauler R. Chemometric tools for image analysis. In: Salzer R, Siesler HW, editors. Infrared and Raman Spectroscopic Imaging. 2nd ed. Weinheim: Wiley; 2014. p. 57–110.

[58] Kong W, Zhang C, Cao F, Liu F, Luo S, Tang Y, He Y. Detection of sclerotinia stem rot on oilseed rape (*Brassica**napus *L.) leaves using hyperspectral imaging. Sensors. 2018;18(6):1764. 10.3390/s18061764. 29857572 10.3390/s18061764 PMC6021932

[59] Sabat-Tomala A, Raczko E, Zagajewski B. Comparison of support vector machine and random forest algorithms for invasive and expansive species classification using airborne hyperspectral data. Remote Sens. 2020;12(3):516. 10.3390/rs12030516.

[60] Saha D, Manickavasagan A. Machine learning techniques for analysis of hyperspectral images to determine quality of food products: a review. Curr Res Food Sci. 2021;4:28–44. 10.1016/j.crfs.2021.01.002. 33659896 10.1016/j.crfs.2021.01.002 PMC7890297

[61] Smith AG, Petersen J, Raghavendra S, Rasmussen CR. Segmentation of roots in soil with U-Net. Plant Methods. 2020;16:13. 10.1186/s13007-020-0563-0. 32055251 10.1186/s13007-020-0563-0 PMC7007677

