# Supplementary material for: Advancing hyperspectral imaging techniques for root systems: a new pipeline for macro- and microscale image acquisition and classification
Source: Plant Methods. 2024 Nov 11;20:171. doi: 10.1186/s13007-024-01297-x (PMC11555864; doi:10.1186/s13007-024-01297-x)
Supplement: Supplementary file 1 — Additional file 1: Supplementary Figure 1. The effect of different apertures on the selection spectra from SAM classifications and K-Means clustering for all data from CONF1. Supplementary Figure 2. The resulting spectra from K-Means clustering in CONF3 at magnification factors of 1.6x and 4x. Supplementary Figure 3. Window size comparison for the second derivative with Savitzy-Golay smoothed root spectra. Supplementary Figure 4. Confusion matrix for random forest models trained on Spectral Angle mapper classifications or K-Means classifications. Supplementary Figure 5. Model predicted image of A. odoratum at 0.63x magnification when the model was trained on all magnification factors. [file 13007_2024_1297_MOESM1_ESM.docx]

**Application of hyperspectral imaging to root system studies: a new pipeline for image acquisition and automated classification for macro- and microscale investigations**

Authors: Corine Faehn^1,*^, Grzegorz Konert^1,2^, Markku Keinänen^1,3,4^, Katja Karppinen^1,5^, Kirsten Krause^1,5^

^1^ Department of Arctic and Marine Biology, The Arctic University of Norway, 9037 Tromsø, Norway

^2^ Department of Life Technologies, University of Turku, 20014 Turku, Finland

^3^ Department of Environmental and Biological Sciences, University of Eastern Finland, 80130 Joensuu, Finland

^4^ Center for Photonics Sciences, University of Eastern Finland, 80110 Joensuu, Finland

^5^ Arctic Centre for Sustainable Energy, The Arctic University of Norway, 9037 Tromsø, Norway

*Correspondence: Corine Faehn corine.a.faehn@uit.no


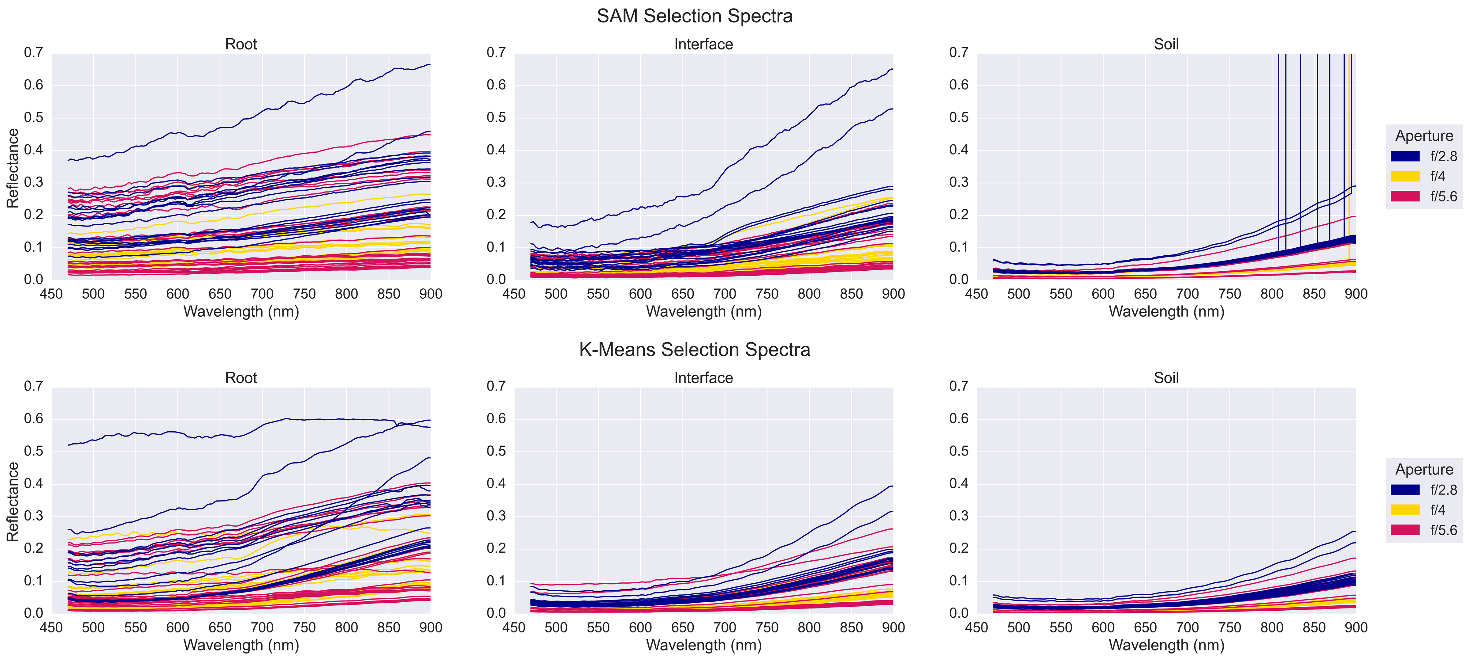


**Supplementary Figure 1.** The effect of different apertures (f/2.8, f/4, or f/5.6) on the selection spectra from SAM classifications and K-Means clustering for all data from CONF1.


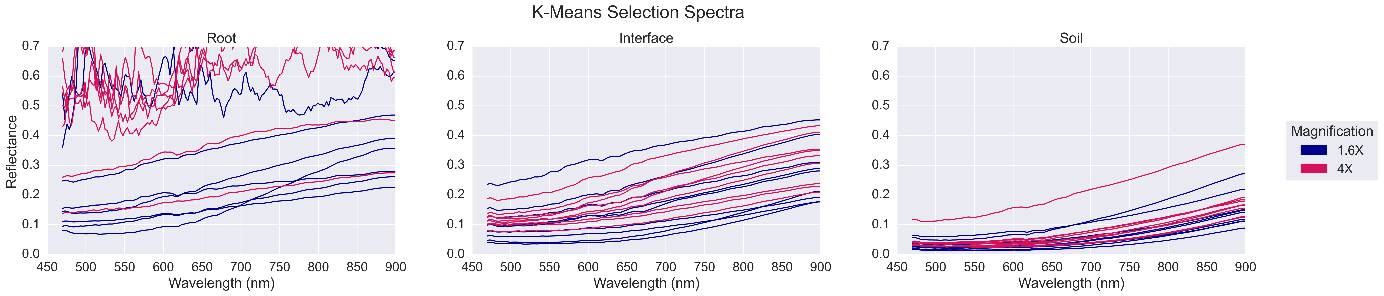


**Supplementary Figure 2.** The resulting spectra from K-Means clustering in CONF3 at magnification factors of 1.6x and 4x.


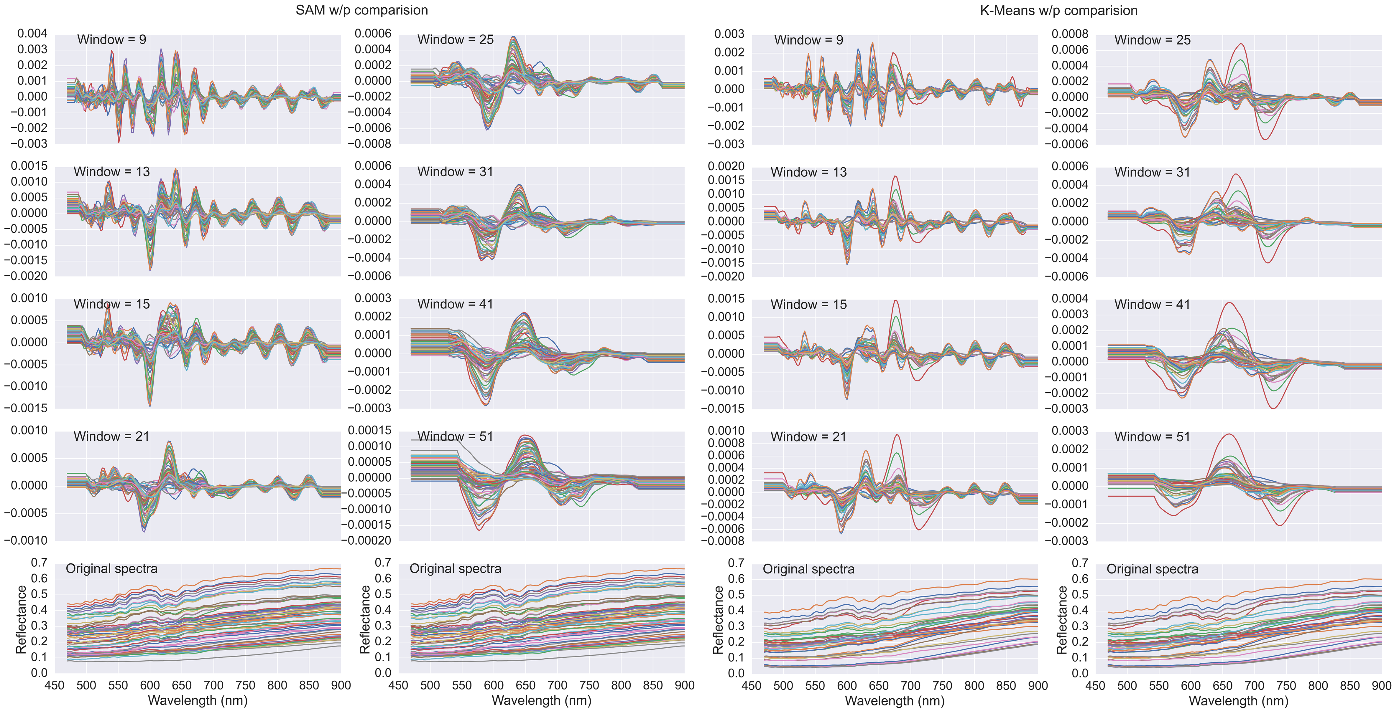


**Supplementary Figure 3.** Window size comparison for the second derivative with Savitzy-Golay (SG) smoothed root spectra.


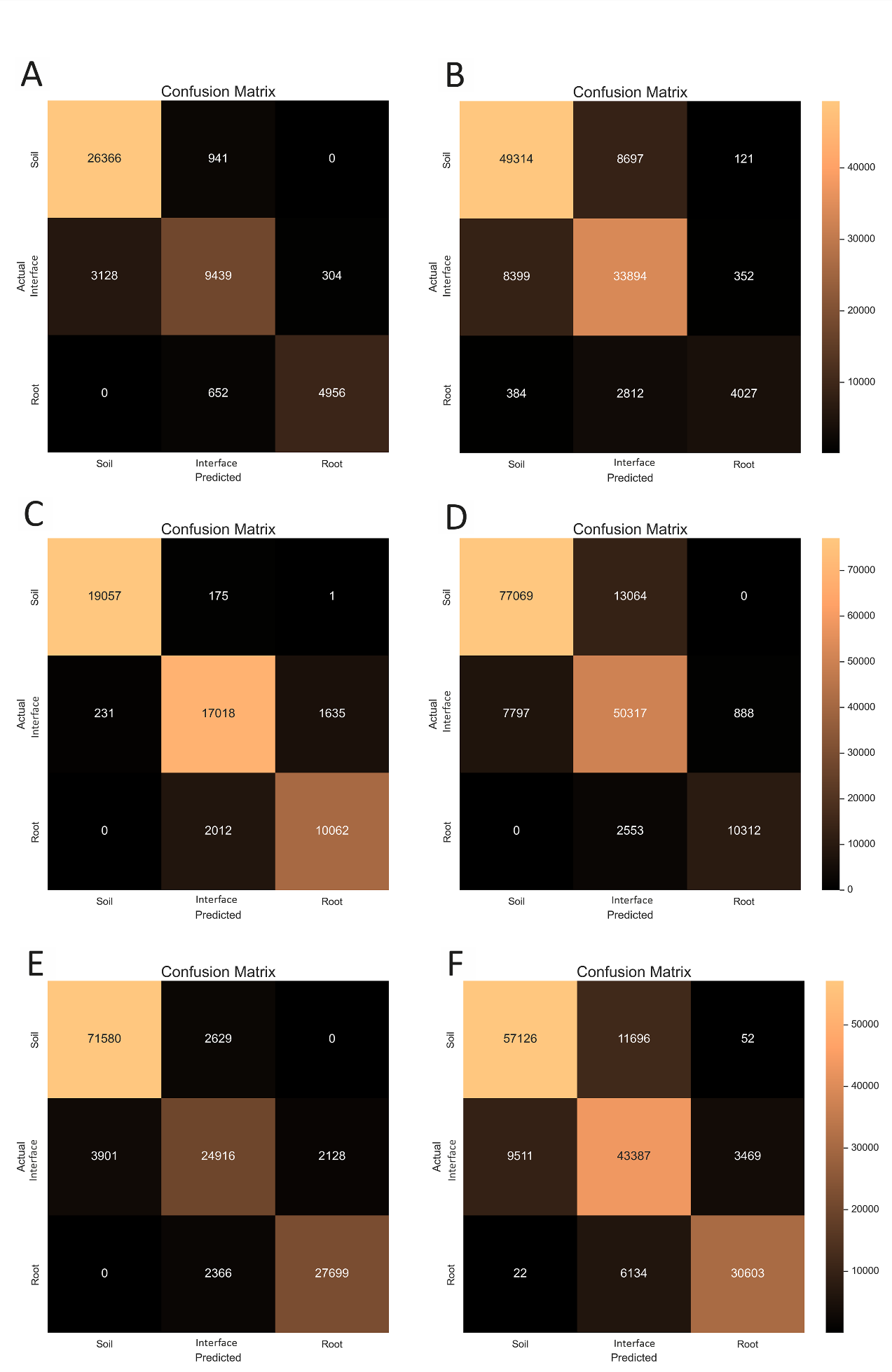


**Supplementary Figure 4.** Confusion matrix for random forest (RF) models trained on Spectral Angle mapper (SAM) classifications (A) CONF1, (C) CONF2, (E), CONF3 or K-Means classifications (B) CONF1, (D) CONF2, (F) CONF3.


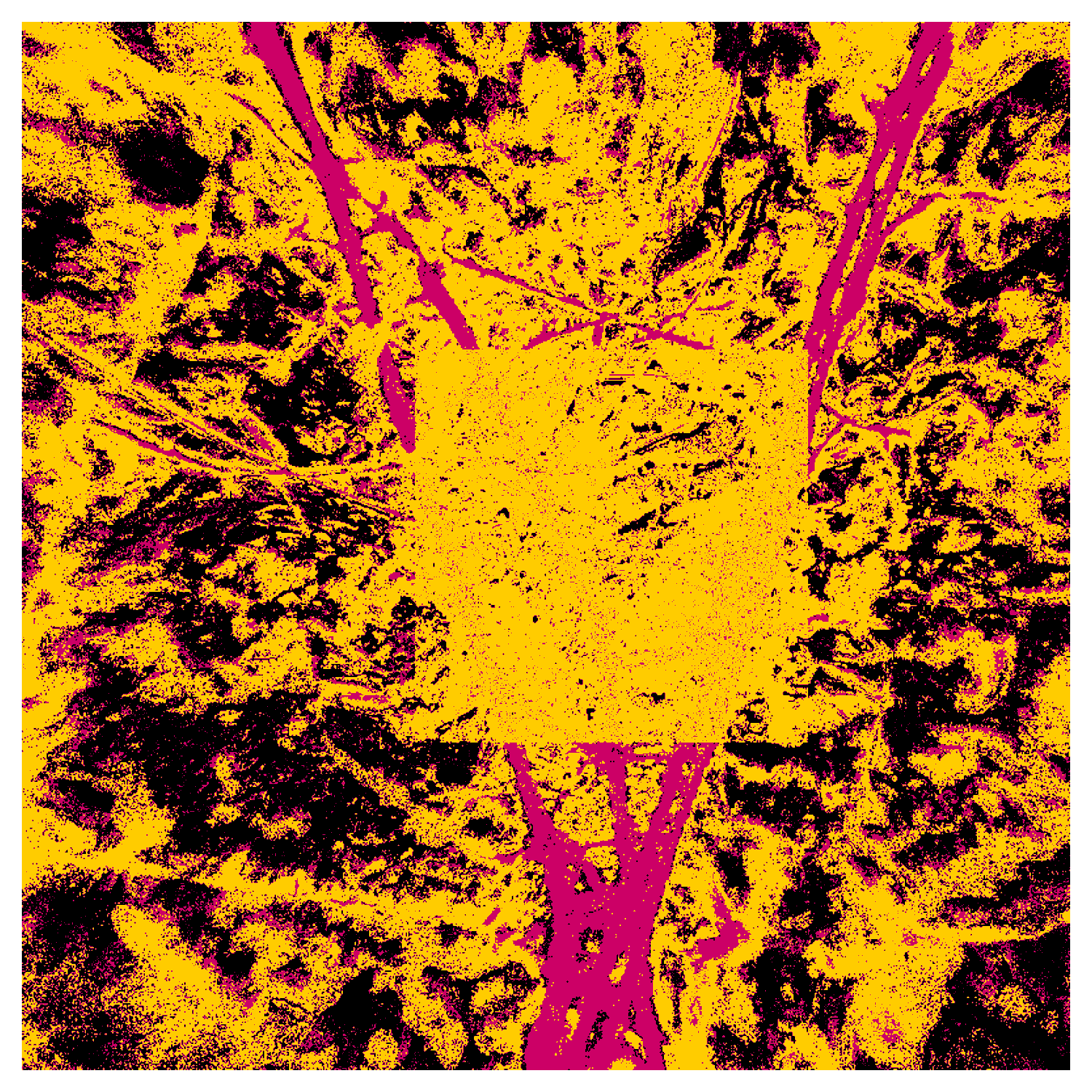


**Supplementary Figure 5.** Model predicted image of *A. odoratum* at 0.63x magnification when the model was trained on all magnification factors (0.63x, 1.6x, and 4x).
